# Risk of New-Onset Hidradenitis Suppurativa in People with Polycystic Ovary Syndrome: a large-scale propensity-score-matched cohort study

**DOI:** 10.7150/ijms.110774

**Published:** 2025-04-22

**Authors:** Shuo-Yan Gau, Chia-Chi Chang, Wei-Ting Hsu, Yen-Ju Chu, Yu-Chiao Ku, Hao Lin, Shiu-Jau Chen, Hui-Chin Chang

**Affiliations:** 1 Department and Graduate Institute of Business Administration, National Taiwan University, Taipei, Taiwan.; 2 Department of Pharmacology, Chung Shan Medical University, Taichung, Taiwan.; 3 Orthopedics Department, Chi-Mei Medical Center, Tainan, Taiwan.; 4 School of Medicine, Chung Shan Medical University, Taichung, Taiwan.; 5 Department of Obstetrics and Gynecology, Kaohsiung Chang Gung Memorial Hospital, Chang Gung University College of Medicine, Kaohsiung 833, Taiwan.; 6 Department of Neurosurgery, Mackay Memorial Hospital, Taipei, Taiwan.; 7 Department of Medicine, Mackay Medical College, New Taipei City, Taiwan.; 8 Evidence-based Medicine Center, Chung Shan Medical University Hospital, Taichung, Taiwan.; 9 Library, Chung Shan Medical University Hospital, Taichung, Taiwan.

**Keywords:** Polycystic Ovary Syndrome, hidradenitis suppurativa, cohort, epidemiology, electronic medical records

## Abstract

**Background:** Hidradenitis suppurativa (HS) is a chronic inflammatory skin disease, while polycystic ovary syndrome (PCOS) is an endocrine disorder. Both conditions share common risk factors, such as androgen excess and obesity. This study aimed to investigate the association between PCOS and the risk of developing HS.

**Method:** A retrospective cohort study was conducted using data from the TriNetX research network, focusing on female patients aged over 18 with PCOS. A control group without PCOS was matched based on age, race, and body mass index using propensity score matching. Hazard ratios (HRs) were calculated to evaluate the risk of HS in PCOS patients across various models.

**Results:** After matching, 141,661 PCOS patients and an equal number of controls were analyzed. PCOS patients showed a significantly increased risk of developing HS (HR: 2.061, 95% confidence interval (CI): 1.910-2.225). The risk remained elevated across different models and sensitivity analyses. Stratified analyses revealed the highest HS risk in younger women (aged 18-39; HR: 2.103, 95% CI: 1.911,2.315), those with a BMI less than 30 (HR: 2.053, 95% CI: 1.761,2.393) and those without diabetes (HR: 1.814, 95% CI: 1.657,1.986).

**Conclusion:** PCOS patients are at a significantly higher risk of developing HS, particularly among younger and more severely affected individuals. Clinical awareness and early detection are essential for managing inflammatory comorbidities in PCOS patients.

## Introduction

As an inflammatory dermatological disorder, hidradenitis suppurativa (HS) can occur in an any area containing folliculo-pilo-sebaceous units (FPSUs) and the predominant region of involvement are the intertriginous skin area such as axillary, groin, anogenital and inframammary regions, as well as other sites prone to friction 1, 2. Incidence of HS varies across different countries, age groups, and genders, with prevalence estimates ranging from less than 1% to 4% 3. Most research identifies females and individuals aged 18 to 29 years as common demographic groups being affected 4. Chronic follicular occlusion further leads to deep-seated inflamed nodule, skin tunnels, open comedones and scar formation 2. The pathogenesis of HS remains incompletely understood; however, it is recognized as a multifactorial condition influenced by various factors, including genetic susceptibility, androgen levels, local immune responses, the composition of the skin microflora, smoking status, and obesity 2, 5-10.

Polycystic ovary syndrome (PCOS) is a heterogenous disorder that involves both endocrine and metabolic aspects in women of reproductive age, and the global prevalence of PCOS has been calculated to be 9.2 % based on a recent meta-analysis 11. Factors involved in the pathogenesis of PCOS includes genetics, elevated luteinizing hormone (LH) levels, obesity, and hyperinsulinemia 12.

The relationship between PCOS and HS has been explored in recent studies 13. The two diseases share certain commonalities, both associated with insulin resistance in pathogenesis and the effectiveness of anti-androgenic drugs in disease management 12, 14, 15. A US-based cross-sectional study reported that people with HS were more than twice as likely to have previous PCOS history 7. Although real-world association has been reported in previous studies, it is still uncertain whether individuals with PCOS are at a higher risk of developing HS. Evidences based on large-scale and longitudinal study that includes more intensive covariates such as age, demographic variables, and BMI levels for PCOS in patients with HS is needed. With further evidence evaluating the real-world association between PCOS and HS, early screening for PCOS comorbidities can be more effectively implemented. The objective of our study is to evaluate the risk of HS development in a large US-based cohort of PCOS patients by comparing them with matched control subjects.

## Methods and Materials

This real-world study was performed in a retrospective cohort design. Study population was extracted from the TriNetX research network. TriNetX is a global database with de-identified access of patients' electronic health records in the collaborative healthcare organizations (HCOs). We utilized the US collaborative network in TriNetX, which focused on population in the United States. It contains data from more than 60 HCOs across the United States and covers more than 80 million patients. This dataset has been frequently used in epidemiological research across multiple clinical disciplines 16.

Female patients aged over 18, with at least two recorded medical visits between January 2005 and December 2017, were selected for further analysis. Among these patients, those diagnosed with polycystic ovary syndrome (PCOS) were categorized into the PCOS cohort. Eligible PCOS cases were identified as patients with greater or equal than two visit records and a documented diagnosis using the International Classification of Diseases, Tenth Revision, Clinical Modification (ICD-10-CM) code E28.2. Those without a prior PCOS diagnosis before the index date were assigned to the healthy control group. Both cohorts excluded any individuals who had a history of hidradenitis suppurativa before the index date or had been diagnosed with any form of cancer, as they were not eligible for further analysis.

All analyses were conducted in the TriNetX analytic platform. In this study, descriptive statistics were used to summarize baseline characteristics of the participants. Continuous variables, such as age at index, were presented as mean ± standard deviation (SD), while categorical variables, including race, comorbidities (e.g., diabetes mellitus, hypertension, hyperlipidemia), socioeconomic status, lifestyle factors (e.g., alcohol dependence, smoking, substance use), medical utilization status, and laboratory data (e.g., body mass index [BMI], C-reactive protein [CRP] levels), were reported as frequencies and percentages (%). To assess differences between the PCOS cohort and the control cohort, standardized mean differences (SMDs) were calculated, with values greater than 0.1 considered indicative of a meaningful imbalance. Furthermore, propensity score matching (PSM) was applied to balance covariates between the two cohorts, adjusting for factors such as age, race, BMI, comorbidities, smoking status, substance use, medical utilization, socioeconomic status, and inflammation-related biomarkers. Information of used administrative codes is depicted in detail in **Table S1**. Each matching instance used a greedy-nearest neighbor method with a caliper width of 0.1. Hazard ratios (HR) were calculated with 95 confidence intervals (95% CI) to evaluate significance of results.

We conducted various sensitivity analyses to assess internal validity. These analyses involved variations in matching criteria, duration of follow-up (5,10 and 15 years after index date), wash-out periods (12,24 and 36 months) and claim-based algorithms (PCOS definition based on inpatient record and frequently used PCOS medications). Additionally, to further examine the impact of hidradenitis suppurativa in PCOS patients, we conducted stratified analyses based on age groups, body mass index (BMI), and the presence of diabetes mellitus.

The TriNetX database was initially approved by the Western Institutional Review Board (Western IRB). In December 2020, a determination made by a qualified expert, as outlined in Section §164.514(b)(1) of the HIPAA Privacy Rule, regarding the de-identification process removed the need for IRB approval in studies utilizing TriNetX.

## Results

### Baseline characteristics of the study subjects

**Figure 1:**
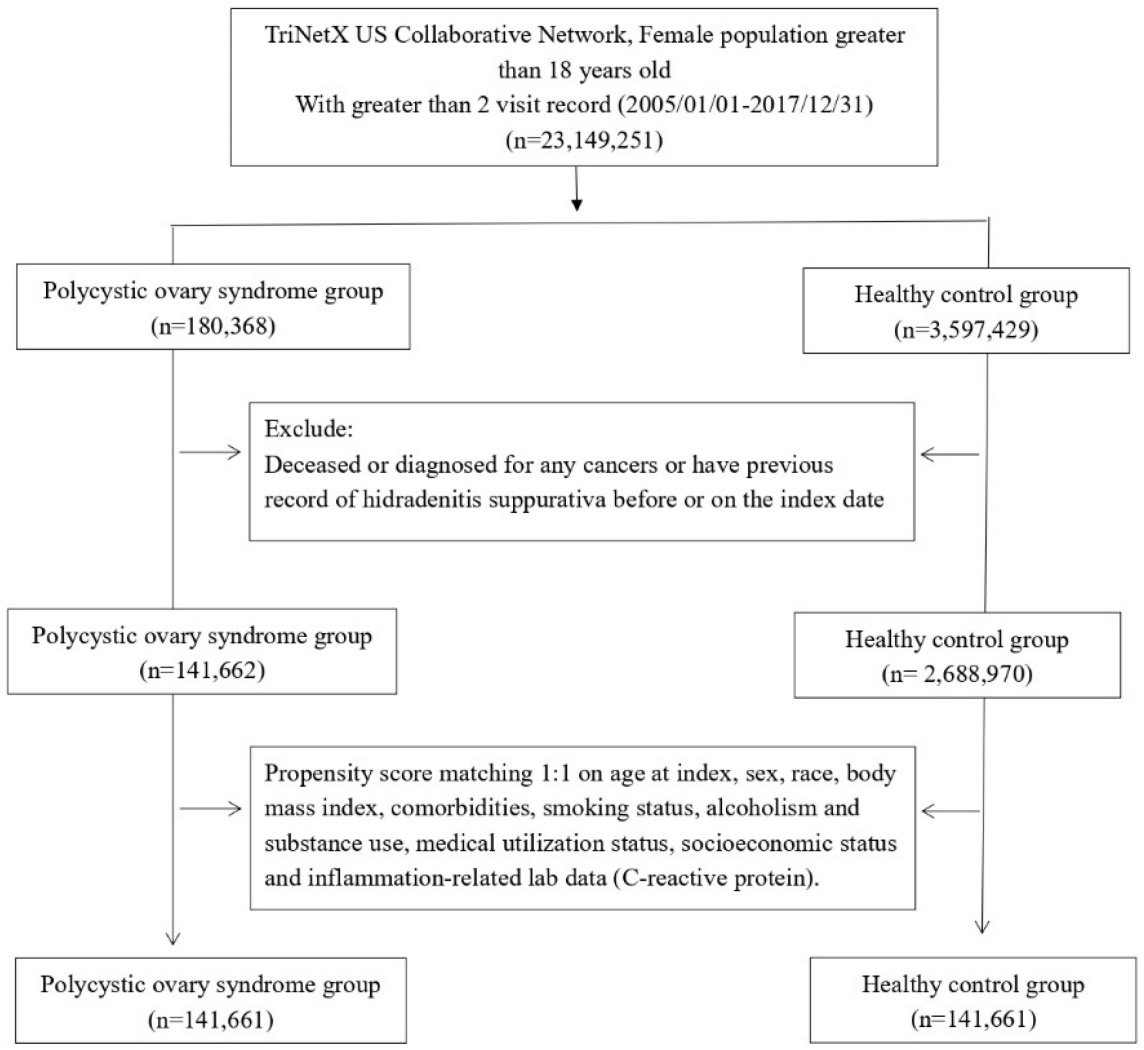
Patient selection process

We evaluated data from 141,661 patients diagnosed with PCOS and an equal number of 141,661 matched controls without PCOS (**Figure 1**). **Table 1** shows the demographic details, comorbidities, socioeconomic factors, lifestyle habits, medical utilization, and lab results for both groups before and after propensity-score matching. Following matching, the PCOS and control groups had similar characteristics, including age, race, comorbidities, lifestyle, socioeconomic status, medical utilization, and laboratory data. The average age of matched participants was 28.4±8.9 years in the PCOS group and 28.5±9.0 years in the control group, with the majority being Caucasian (64.3% in both cohorts).

### Risk of developing hidradenitis suppurativa in patients with polycystic ovary syndrome in different models

**Figure 2:**
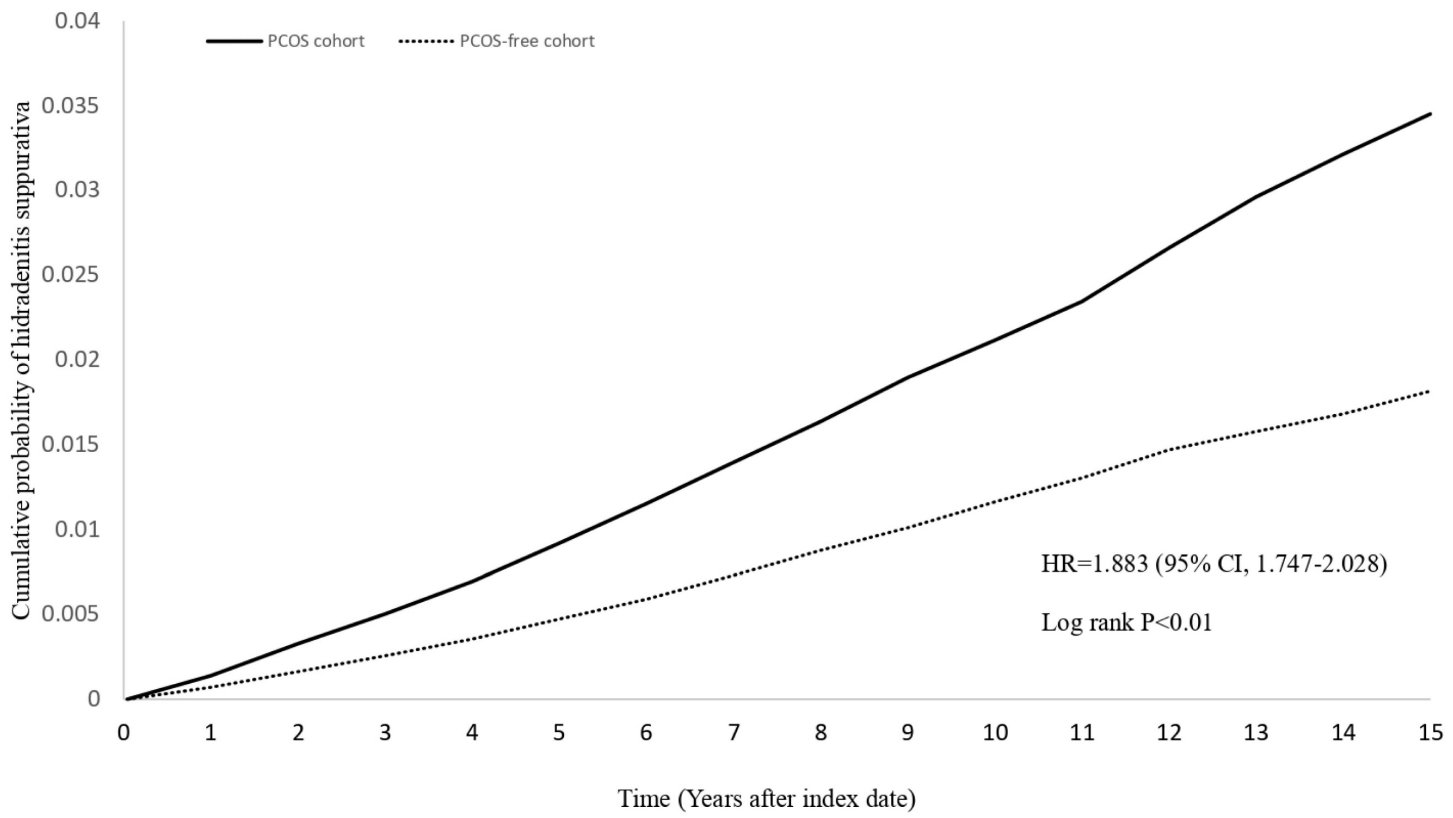
Kaplan-Meier plot of new-onset HS

Risk of HS for the PCOS cohort were 3.089 (95% CI=2.944-3.240) for crude model without performing propensity score matching, 2.186 (95% CI=2.021-2.364) for covariates of propensity score matching including age at index, sex and race, and 2.061 (95% CI=1.91-2.225) for covariates of propensity score matching including age at index, sex, race and BMI (**Figure 2**, **Table 2**). The hazard ratios (HR) for developing HS in the PCOS cohort were 1.896 (95% CI: 1.756-2.047), 1.895 (95% CI: 1.750-2.052), and 1.921 (95% CI: 1.768-2.088) for 12-, 24-, and 36-month wash-out periods, respectively. For follow-up periods of 5, 10, and 15 years, the HRs were 2.001 (95% CI: 1.797-2.228), 1.865 (95% CI: 1.723-2.018), and 1.883 (95% CI: 1.747-2.028), respectively. Additionally, the HR for HS was 1.563 (95% CI: 1.209-2.019) for patients diagnosed with PCOS with more than two visit records and prescribed related medications (clomiphene, letrozole). For patients with more than two inpatient visits due to PCOS, the HR was 2.232 (95% CI: 1.963-2.539).

### Stratified analyses by age, BMI and Diabetes Mellitus

As detailed in **Table 3**, the HR for females aged 18-39 and 40-64 was 2.103 (95% CI: 1.911-2.315) and 1.671 (95% CI: 1.477-1.891), respectively. For females aged 65 and older, the HR was 1.046 (95% CI: 0.262-4.184). The HR for females with a BMI over or under 30 was 1.468 (95% CI: 1.347-1.598) and 2.053 (95% CI: 1.761-2.393), respectively. The HR for females with and without diabetes mellitus was 1.503 (95% CI: 1.322-1.710) and 1.814 (95% CI: 1.657-1.986), respectively.

## Discussion

Both hidradenitis suppurativa (HS) and polycystic ovary syndrome (PCOS) are marked by chronic inflammation and share common risk factors such as obesity and insulin resistance, with both conditions responding to anti-androgen therapies. The association between these diseases may be driven by shared pathogenic mechanisms, particularly androgen excess and metabolic dysfunction. A systematic review of five case-control studies revealed that women with HS are at a significantly increased risk of developing PCOS compared to PCOS-free controls, with an odds ratio of 2.64 (95% CI: 1.69-4.11) 8. Moreover, studies have highlighted the comorbidity between HS and PCOS, recommending that women with HS exhibiting symptoms of androgen excess should be screened for PCOS 17, 18. Despite extensive research on the prevalence of PCOS among women with HS, there remains a significant gap in studies examining the incidence of HS among patients with PCOS. In the current study, we report a significantly increased risk of developing HS in individuals with PCOS in different models. This association underscores the strong relationship between these two conditions and highlights the importance of considering HS risk in patients with PCOS.

One of the key features of PCOS is elevated androgen levels, which can lead to increased sebum production, promoting follicular occlusion and inflammation—hallmarks of HS. Additionally, both PCOS and HS are associated with systemic chronic inflammation; patients with PCOS typically show elevated levels of CRP and other inflammatory markers, a pattern also observed in HS. This suggests that these two conditions may share common inflammatory mechanisms 2. Furthermore, insulin resistance and obesity, common in PCOS, may exacerbate the development of HS. The high prevalence of comorbidities such as obesity, type 2 diabetes, and metabolic syndrome in both PCOS and HS further strengthens the potential relationship between these disorders.

In our study, we examined the association between PCOS and the risk of developing HS using three models with varying levels of adjustment. Despite slight reductions in the association after accounting for factors such as BMI, the results consistently showed that PCOS patients have more than double the risk of developing HS compared to controls. This finding is consistent with previous research by Garg et al. 7, further supporting the independent relationship between PCOS and HS. Sensitivity analyses, including variations in wash-out periods and follow-up durations, consistently demonstrated a significantly elevated cumulative risk of HS in PCOS patients, reinforcing the temporal link between these conditions.

The predominance of HS in women, its onset after puberty, premenstrual flare-ups, and improvement during pregnancy strongly suggest a hormonal influence, though the exact mechanisms remain elusive 19. Hyperandrogenism, a common feature of PCOS, as androgen excess is frequently observed in HS patients, even in those without a formal PCOS diagnosis, manifesting as acne, hirsutism, irregular menstruation, and infertility. This is corroborated by elevated free androgen index and low SHBG levels 20. However, studies by Barth et al. (1996) 21 and Harrison et al. (1988) 22 found no significant differences in testosterone or DHEAS levels between HS patients and controls, suggesting that hyperandrogenism may not always be systemically evident. Instead, local androgen production in the skin might play a role in HS, as the skin can synthesize sex hormones via intracrine or paracrine mechanisms, potentially contributing to HS pathology 23, 24. In PCOS, systemic hyperandrogenism could disrupt local skin hormone balance, leading to key features of HS such as infundibular hyperkeratosis, follicular hyperplasia, and perifolliculitis 25, 26.

Meta-inflammation, a chronic low-grade inflammatory state which often linked to obesity, plays a critical role in both PCOS and HS. This condition generally presents by increased levels of pro-inflammatory cytokines. These cytokines not only drive systemic inflammation but also contribute to the development of HS by promoting a pro-inflammatory environment that affects skin homeostasis. The excessive adipose tissue present in individuals with obesity, along with an imbalanced gut microbiome, is believed to drive this persistent inflammatory response, leading to the upregulation of these pro-inflammatory molecules 27, 28. Meta-inflammation not only exacerbates the symptoms of PCOS and HS but also promotes the development of comorbidities like insulin resistance and type 2 diabetes, which are common to both conditions. These findings suggest that PCOS and HS may share potential inflammatory pathways, linking their pathogenesis through systemic inflammatory processes.

In our analysis, the risk of developing HS was notably higher when using claim-based algorithms to classify PCOS patients by treatment patterns (**Table 2**, models 1j and 2k). Model 1j, capturing patients with over two outpatient visits and related prescriptions, primarily reflected mild to moderate PCOS cases. Conversely, Model 2k, encompassing patients with more than two hospitalizations, represented more severe cases. The hazard ratio (HR) for HS in the 2k model was 2.232 (95% CI: 1.963, 2.539), significantly surpassing the HR of 1.563 (95% CI: 1.209, 2.019) in the 1j model. The 2k model likely includes patients with severe manifestations of PCOS, which may exacerbate chronic inflammation, thereby increasing the risk of developing HS.

In our stratification analysis, we observed a notable increase in HS risk among individuals aged 18-64 with PCOS, while no substantial difference was evident in those aged 65 and above compared to their non-PCOS counterparts. This age-related risk disparity can be attributed to several factors. Both PCOS and HS typically manifest earlier in life, with HS symptoms frequently appearing between puberty and age 40^4^. Similarly, PCOS primarily affects women of reproductive age. Besides, the lack of increased risk in the older cohort (65+ years) may be explained by significant post-menopausal hormonal changes. The drastically altered endocrine environment in post-menopausal women might influence HS incidence. This observation aligns with existing literature, which indicates that HS onset after menopause is extremely rare 29. Moreover, aging is associated with immunosenescence, characterized by a general decline in immune function, particularly in inflammatory responses. This age-related reduction in inflammation may contribute to the lower HS risk observed in older individuals with PCOS. Furthermore, we included BMI and diabetes status as control variables in our analysis. The results consistently showed that, regardless of diabetes status or whether BMI was greater than or equal to 30, individuals with PCOS exhibited a higher risk of developing HS compared to those without PCOS. This suggests that the association between PCOS and HS is robust across these variables, further emphasizing the need for targeted clinical awareness and intervention.

Our study benefits from several methodological strengths, though certain limitations are acknowledged. The use of the TriNetX database allowed for a large sample size, improving both the statistical power and the generalizability of the results. To reduce confounding bias, propensity score matching was applied, ensuring balanced baseline characteristics between the PCOS and control groups. Furthermore, sensitivity analyses were conducted to validate the consistency of our findings under different analytical conditions. However, some limitations should be considered. Our reliance on administrative data may have resulted in incomplete or inaccurate diagnoses of HS and other variables, introducing potential misclassification bias. Furthermore, while the TriNetX database draws from a wide range of healthcare organizations, it may not fully capture the diversity of the broader population, potentially leading to selection bias. Another notable limitation is the absence of differentiation between the various PCOS phenotypes as defined by the Rotterdam criteria 30. Each phenotype presents distinct clinical characteristics that may influence the risk of developing HS, but our study did not conduct a detailed analysis of HS incidence across these PCOS phenotypes. Such analysis could provide deeper insights into the pathogenic relationship between PCOS and HS. Future research should explore these phenotypic variations to better understand the connection between these conditions.

In conclusion, our findings provide evidences that the likelihood of developing HS is significantly higher in patients with PCOS, especially among those of reproductive age and individuals with more severe or clinically confirmed cases of PCOS. These findings emphasize the importance of increased awareness, early detection, and a collaborative, multidisciplinary approach in the prevention of inflammatory comorbidities in PCOS patients.

## Supplementary Material

Supplementary table.

## Figures and Tables

**Table 1:** Baseline characteristics

	Before matching			After matching^a^		
	Polycystic ovary syndrome cohort (n=141,662)	Control cohort (n= 2,688,970)	SMD	Polycystic ovary syndrome cohort (n=141,661)	Control cohort (n=141,662)	SMD
**Age at index**						
Mean ± SD	28.4 ± 8.9	38.2 ± 21.1	**0.61**	28.4 ± 8.9	28.5 ± 9.0	0.01
**Race, n (%)**						
White	91064(64.3)	1721150(64.0)	0.01	91063(64.3)	91049(64.3)	0.00
Black or African American	16885(11.9)	434056(16.1)	**0.12**	16885(11.9)	16896(11.9)	0.00
Asian	5697(4.0)	93105(3.5)	0.03	5697(4.0)	6282(4.4)	0.02
Native Hawaiian or Other Pacific Islander	582(0.4)	11047(0.4)	0.00	582(0.4)	439(0.3)	0.02
American Indian or Alaska Native	503(0.4)	8414(0.3)	0.01	503(0.4)	538(0.4)	0.00
**Comorbidities, n (%)**						
Essential hypertension	7017(5.0)	287203(10.7)	**0.21**	7017(5.0)	6725(4.7)	0.01
Hyperlipidemia	3973(2.8)	183861(6.8)	**0.19**	3973(2.8)	3650(2.6)	0.01
Diabetes mellitus	5427(3.8)	116683(4.3)	0.03	5426(3.8)	5437(3.8)	0.00
Anxiety	11007(7.8)	175140(6.5)	0.05	11006(7.8)	10357(7.3)	0.02
Depression	8609(6.1)	137705(5.1)	0.04	8608(6.1)	7596(5.4)	0.03
Schizophrenia	158(0.1)	4739(0.2)	0.02	158(0.1)	202(0.1)	0.01
Suicide attempt	30(0.0)	437(0.0)	0.00	30(0.0)	42(0.0)	0.01
Systemic lupus erythematosus	253(0.2)	7235(0.3)	0.02	253(0.2)	369(0.3)	0.02
Crohn's disease	251(0.2)	5268(0.2)	0.00	251(0.2)	295(0.2)	0.01
Ulcerative colitis	167(0.1)	4273(0.2)	0.01	167(0.1)	181(0.1)	0.00
Ankylosing spondylitis	39(0.0)	783(0.0)	0.00	39(0.0)	31(0.0)	0.00
Rheumatoid arthritis	30(0.0)	1848(0.1)	0.02	30(0.0)	23(0.0)	0.00
Chronic kidney disease	370(0.3)	25597(1.0)	0.09	370(0.3)	536(0.4)	0.02
**Socioeconomic status, n (%)**						
Socioeconomic/psychosocial circumstances problem	1278(0.9)	23136(0.9)	0.00	1277(0.9)	1283(0.9)	0.00
**Lifestyle, n (%)**						
Alcohol dependence, smoking and substance use	4563(3.2)	83716(3.1)	0.01	4562(3.2)	4630(3.3)	0.00
**Medical Utilization Status, n (%)**						
Ambulatory visit	78637(55.5)	1566571(58.3)	0.06	78637(55.5)	78538(55.4)	0.00
Inpatient visit	13731(9.7)	337432(12.5)	0.09	13731(9.7)	13710(9.7)	0.00
**Laboratory data**						
BMI, n (%)						
≥ 25 (kg/m^2^)	31008(21.9)	505080(18.8)	0.08	31007(21.9)	31141(22.0)	0.00
CRP, n (%)						
≥ 10 (mg/L)	1571(1.1)	26682(1.0)	0.01	1571(1.1)	1597(1.1)	0.00

Bold font represents a standardized difference was more than 0.1 PCOS, Polycystic ovary syndrome; HS: Hidradenitis Suppurativa; SMD, standardized mean difference ^a^ Propensity score matching was used. The covariates included age at index, sex, race, body mass index, comorbidities (such as diabetes mellitus, hypertension, hyperlipidemia, Crohn's disease, ulcerative colitis, ankylosing spondylitis, and rheumatoid arthritis), smoking status, alcoholism and substance use (mental and behavioral disorders due to psychoactive substance use), medical utilization status, socioeconomic status (issues related to housing and economic conditions, potential health hazards related to socioeconomic and psychosocial factors), and inflammation-related lab data (C-reactive protein).

**Table 2:** Hazard ratio of hidradenitis suppurativa with 95% confidence interval under various models.

Various matching covariates	Model 1^a^	Model 2^b^	Model 3^c^
Non-PCOS controls	1.00	1.00	1.00
PCOS patients	**3.089 (2.944,3.240)**	**2.186 (2.021,2.364)**	**2.061 (1.910,2.225)**
**Various wash-out periods**	**Model 1^d^**	**Model 2^e^**	**Model 3^f^**
Non-PCOS controls	1.00	1.00	1.00
PCOS patients	**1.896 (1.756,2.047)**	**1.895 (1.750,2.052)**	**1.921 (1.768,2.088)**
**Various follow-up times**	**Model 1^g^**	**Model 2^h^**	**Model 3^i^**
Non-PCOS controls	1.00	1.00	1.00
PCOS patients	**2.001 (1.797,2.228)**	**1.865 (1.723,2.018)**	**1.883 (1.747,2.028)**
**Various claim-based algorithms**	**Model 1^j^**	**Model 2^k^**	
Non-PCOS controls	1.00	1.00	
PCOS patients	**1.563 (1.209,2.019)**	**2.232 (1.963,2.539)**	

PCOS, polycystic ovary syndrome; HS, hidradenitis suppurativa. In this table, apart from the analyses of varied matching covariates, propensity score matching was used in all analyses. The covariates included age at index, sex, race, body mass index, comorbidities (such as diabetes mellitus, hypertension, hyperlipidemia, Crohn's disease, ulcerative colitis, ankylosing spondylitis, and rheumatoid arthritis), smoking status, alcoholism and substance use (mental and behavioral disorders due to psychoactive substance use), medical utilization status, socioeconomic status (issues related to housing and economic conditions, potential health hazards related to socioeconomic and psychosocial factors), and inflammation-related lab data (C-reactive protein). ^a^ Crude model without performing propensity score matching. ^b^ Covariates of propensity score matching includes age at index, sex, race. ^c^ Covariates of propensity score matching includes age at index, sex, race, BMI. ^d^ Wash-out period was set as 12 months in this model. Incident hidradenitis suppurativa occurred within 12 months were not calculated as outcome events. ^e^ Wash-out period was set as 24 months in this model. Incident hidradenitis suppurativa occurred within 24 months were not calculated as outcome events. ^f^ Wash-out period was set as 36 months in this model. Incident hidradenitis suppurativa occurred within 36 months were not calculated as outcome events. ^g^ Follow-up period was set as 5 years in this model. ^h^ Follow-up period was set as 10 years in this model. ^i^ Follow-up period was set as 15 years in this model. ^j^ Only patients being diagnosed of Polycystic ovary syndrome with more than 2 visit records and with the prescription of related medications (clomiphene, letrozole) were included as Polycystic ovary syndrome group in this model. ^k^ Only patients with more than 2 impatient visit record due to Polycystic ovary syndrome were included as Polycystic ovary syndrome group in this model.

**Table 3:** Stratification analysis of hidradenitis suppurativa risk in polycystic ovary syndrome (PCOS) patients in 15-year follow-up

	Cases occurring new-onset hidradenitis suppurativa		
Subgroups	PCOS cohort No. of outcome event (%)	Control cohort No. of outcome event (%)	HR (95% CI)^a^
**Age at index date**			
18-39 years old	1266(1.7)	623(0.8)	**2.103 (1.911,2.315)**
40-64 years old	653(1.1)	408(0.7)	**1.671 (1.477,1.891)**
≥ 65 years old	≤10 (≤0.1)	≤10 (≤0.1)	1.046 (0.262,4.184)
**BMI ≥ 30**			
Yes	1206(2.1)	935(1.6)	**1.468 (1.347,1.598)**
No	468(0.8)	251(0.4)	**2.053 (1.761,2.393)**
**Diabetes Mellitus**			
With	563(2.3)	396(1.6)	**1.503 (1.322,1.710)**
Without	1253(1.1)	745(0.7)	**1.814 (1.657,1.986)**

^a^ Propensity score matching was used in all analyses. The covariates included age at index, sex, race, body mass index, comorbidities (such as diabetes mellitus, hypertension, hyperlipidemia, Crohn's disease, ulcerative colitis, ankylosing spondylitis, and rheumatoid arthritis), smoking status, alcoholism and substance use (mental and behavioral disorders due to psychoactive substance use), medical utilization status, socioeconomic status (issues related to housing and economic conditions, potential health hazards related to socioeconomic and psychosocial factors), and inflammation-related lab data (C-reactive protein).
