# Supplementary material for: Risk of New-Onset Hidradenitis Suppurativa in People with Polycystic Ovary Syndrome: a large-scale propensity-score-matched cohort study
Source: Int J Med Sci. 2025 Apr 22;22(10):2269–76. doi: 10.7150/ijms.110774 (PMC12080565; doi:10.7150/ijms.110774)
Supplement: Supplementary file 1 — Supplementary table. [file ijmsv22p2269s1.pdf]

Supplementary Files

Supplementary Tables

Table S1. Utilized proxy codes [a]

Description	ICD-10-CM codes
Polycystic ovary syndrome	E28.2
Hidradenitis Suppurativa	L73.2
Neoplasms	C00-D49
Essential hypertension	I10
Hyperlipidemia	E78.5
Diabetes mellitus	E08-E13
Anxiety	F40-F48
Depression	F32
Schizophrenia	F20
Suicide attempt	T14.91
Systemic lupus erythematosus	M32
Crohn's disease	K50
Ulcerative colitis	K51
Ankylosing spondylitis	M45
Rheumatoid arthritis	M05
Chronic kidney disease	N18
Encounter for general examination	Z00

[a]ICD-10-CM: International Classification of Diseases, Tenth Revision, Clinical Modification
